# Crystal Structure of HIV-1 gp41 Including Both Fusion Peptide and Membrane Proximal External Regions

**DOI:** 10.1371/journal.ppat.1000880

**Published:** 2010-05-06

**Authors:** Victor Buzon, Ganesh Natrajan, David Schibli, Felix Campelo, Michael M. Kozlov, Winfried Weissenhorn

**Affiliations:** 1 Unit of Virus Host Cell Interactions (UVHCI) UMI 3265 Université Joseph Fourier-EMBL-CNRS, Grenoble, France; 2 Department of Physiology and Pharmacology, Sackler Faculty of Medicine, Tel Aviv University, Tel Aviv, Israel; Harvard Medical School, United States of America

## Abstract

The HIV-1 envelope glycoprotein (Env) composed of the receptor binding domain gp120 and the fusion protein subunit gp41 catalyzes virus entry and is a major target for therapeutic intervention and for neutralizing antibodies. Env interactions with cellular receptors trigger refolding of gp41, which induces close apposition of viral and cellular membranes leading to membrane fusion. The energy released during refolding is used to overcome the kinetic barrier and drives the fusion reaction. Here, we report the crystal structure at 2 Å resolution of the complete extracellular domain of gp41 lacking the fusion peptide and the cystein-linked loop. Both the fusion peptide proximal region (FPPR) and the membrane proximal external region (MPER) form helical extensions from the gp41 six-helical bundle core structure. The lack of regular coiled-coil interactions within FPPR and MPER splay this end of the structure apart while positioning the fusion peptide towards the outside of the six-helical bundle and exposing conserved hydrophobic MPER residues. Unexpectedly, the section of the MPER, which is juxtaposed to the transmembrane region (TMR), bends in a 90°-angle sideward positioning three aromatic side chains per monomer for membrane insertion. We calculate that this structural motif might facilitate the generation of membrane curvature on the viral membrane. The presence of FPPR and MPER increases the melting temperature of gp41 significantly in comparison to the core structure of gp41. Thus, our data indicate that the ordered assembly of FPPR and MPER beyond the core contributes energy to the membrane fusion reaction. Furthermore, we provide the first structural evidence that part of MPER will be membrane inserted within trimeric gp41. We propose that this framework has important implications for membrane bending on the viral membrane, which is required for fusion and could provide a platform for epitope and lipid bilayer recognition for broadly neutralizing gp41 antibodies.

## Author Summary

HIV-1 employs its envelope glycoprotein complex (Env) composed of gp120 and gp41 to catalyze cell entry. Both Env subunits undergo conformational changes triggered by the gp120-mediated interactions with cellular receptors. Notably, gp41 refolds into a core six-helical bundle structure which is central to the fusion process. Here we report the structural basis for the folding of the linker regions connecting to the membrane anchors of gp41, namely to the transmembrane region (MPER) and to the fusion peptide (FPPR). Our structural analysis shows helical assemblies of FPPR and MPER which increase the melting temperature of gp41 and position the fusion peptide towards the outside of the six-helix bundle structure at this stage of gp41 refolding. It suggests that part of MPER must be inserted into the viral membrane, which would induce membrane curvature as postulated to be required for the fusion reaction. Thus our findings shed new light on the refolding of gp41, which contributes energy to the fusion reaction and reveals for the first time the structural principles of MPER membrane interaction within trimeric gp41. We propose that the structure presents a late fusion intermediate state that provides a new framework for fusion inhibitor development and MPER immunogen design.

## Introduction

HIV-1 employs its trimeric env glycoprotein, composed of the receptor binding domain gp120 and the membrane anchored fusion protein subunit gp41 to enter host cells. Gp120 interacts sequentially with its cellular receptors CD4 and coreceptor CCR5 or CXCR4 [1], which induce a cascade of conformational changes in gp120 and gp41 [2], [3]. As a consequence the core of gp41 folds into a six helical bundle structure that leads to the apposition of viral and cellular membranes [4], [5].

Gp41 catalyses membrane fusion and current models suggest that receptor binding leads to the exposure of the gp41 fusion peptide (FP), which interacts with the target cell membrane producing an intermediate, pre-hairpin state bridging two membranes. This pre-hairpin has a relatively long half-life [6] and constitutes the target for inhibitory peptides [7], [8], [9] and neutralizing antibodies directed against HR1 [10] [11] and MPER [12], [13]. Potentially at this stage, MPER was hypothesized to be membrane embedded based on the reactivity of broadly neutralizing MPER-specific antibodies [14], [15], [16], [17], [18]. The pre-hairpin then refolds into the six-helix bundle core structure [4], [5] and it is this transition that catalyzes membrane fusion [19]. Six-helix bundle core formation is achieved before fusion pore opening [20]. Experimental evidence [6], [19], [21] suggest that fusion proceeds via lipidic intermediate states, a membrane stalk, opening of the fusion pore and its expansion [22]. Mutagenesis analyses indicate that both linkers to the membrane anchors, FPPR and MPER, are implicated in fusion [23], [24] and the TMRs play an important role in fusion pore enlargement [22], [25], [26].

The energy released during gp41 refolding is used to overcome the kinetic barrier [3], [27], which is underlined by the high thermostability of gp41 core structures [28], [29] constituting a common feature of viral fusion proteins [30] [31] [32] [26]. Although the free energy liberated during refolding of one trimer might be sufficient for fusion [26] consistent with experimental evidence [33], other studies imply that cooperativity of several trimers is required [34].

In order to understand the structural basis of MPER and FPPR in the context of gp41 trimers and their potential contribution to stabilize the gp41 post fusion conformation, we have assembled gp41 containing FPPR and MPER (gp41_528–683_). Thermostability measurements show that inclusion of FPPR and MPER increases the melting temperature (T_m_) substantially compared to the gp41 core, suggesting that the gain of free energy can be directly coupled to membrane fusion. The crystal structure of gp41_528–683_ shows helical refolding of FPPR and part of MPER as well as the potential membrane insertion of MPER adjacent to the TMR. The structure thus indicates for the first time that part of MPER can insert into the viral membrane within trimeric gp41 and supports the hypothesis that a number of neutralizing gp41 antibodies recognize MPER in a membrane environment.

## Results

### Thermal denaturation of the extracellular domain of gp41

**Figure 1:**
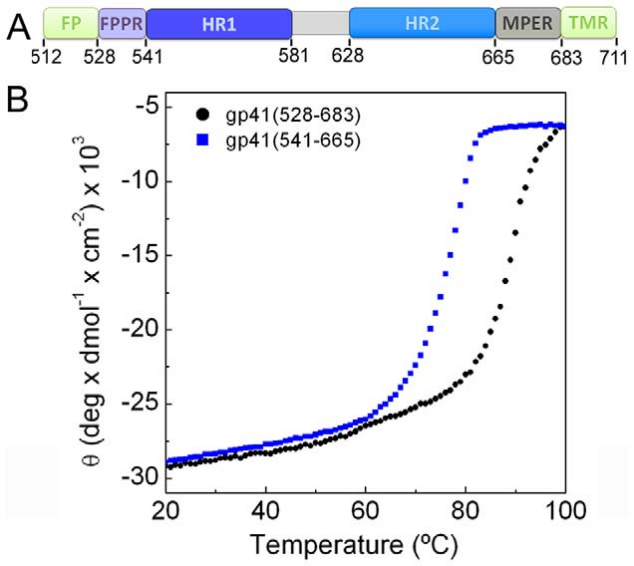
FPPR and MPER increase the melting temperature of gp41. A) Schematic overview of gp41; FP, fusion peptide; FPPR, fusion peptide proximal region; HR1, heptad repeat 1; HR2, heptad repeat 2; MPER, membrane proximal external region; TMR, transmembrane region. B) Unfolding of gp41_528–683_ and gp41_541–665_ monitored by circular dichroism spectroscopy at 222 nm.

We assembled the extracellular domain of gp41 from two fragments containing residues 528 (lacking 16 N-terminal gp41 residues including FP) to 581 (FPPR-heptad repeat 1, HR1) and residues 629 to 683 (HR2-MPER) (gp41_528–683_) (Fig. 1A and Fig. S1). Both chains contain N-terminal Flag-tags to produce a soluble and monodisperse complex (Fig. S2). Circular dichroism analysis reveals a high helical content of ∼90% (Fig. S3A) and a melting temperature (T_m_) of 87.6°C (Fig. 1B). In comparison, the core fragment of gp41 composed of HR1 and HR2 [5] (gp41_541–665_) containing N-terminal Flag-tags shows a T_m_ of 75.1°C (Fig. 1B). Thus FPPR and MPER interact and impart most likely increased trimer stability.

### Crystal structure of gp41 and MPER membrane insertion

**Figure 2:**
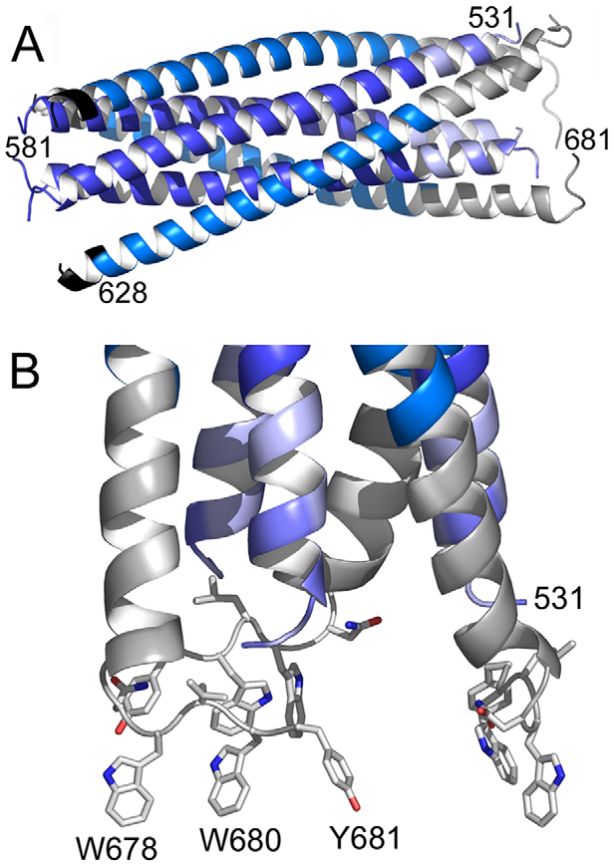
Crystal structure of gp41_528–683_ reveals a 90 Å long rod-like structure. A) Ribbon representation of gp41. The previously determined core is colored dark blue (HR1) and marine blue (HR2). The flag sequence present at the N-terminus of HR2 is shown in black. FPPR is colored in light blue and MPER in grey. Note that the N-terminus of FPPR (residue 531) points towards the outside of the rod. B) Close up of the MPER and FPPR region shows the exposure of aromatic side chains Trp 678, Trp 680 and Tyr 681 towards the membrane.

Gp41_528–683_ was crystallized in space group P6_3_. The structure was solved by molecular replacement and refined to a resolution of 2 Å (Table 1). The crystal structure composed of residues 531–581 and 629–681 plus 5 N-terminal Flag-tag residues reveals the six helical bundle core [4], [5] with FPPR and MPER extending in a helical conformation resulting in an 88 Å-long rod-like structure (Fig. 2A). A striking feature of the structure is a ∼90° turn of the MPER chain at Asn 677 which positions the remaining residues including Trp 678, Trp 680 and Tyr 681 perpendicular to the rod (Fig. 2B). Two disordered C-terminal residues must connect gp41 into the TMR in the membrane (Fig. S4). As a consequence, Trp 678, Trp 680 and Tyr 681 are exposed towards the membrane and well positioned to insert their side chains into the bilayer (Fig. S4). In order to calculate the membrane curvature generated by a shallow embedding of these MPER residues into the outer leaflet of a bilayer, we used a model for membrane bending by hydrophobic insertions [35]. This suggests that one gp41 chain produces local curvature of ∼0.65 nm^−1^; thus a gp41 trimer might stabilize a membrane cylinder of about 15 nm diameter, which would facilitate fusion considerably [36].

**Table 1:** Crystallographic statistics.

Data collection statistics	
Resolution(Å)	91.00 - 2.00 (2.11 – 2.00)
Completeness(%)	96.5 (84.0)
I/σ(I)	17.9 (2.8)
R_merge_	0.065 (0.331)
Redundancy	5.9 (2.4)
Number of reflections	129,367 (6627)
Unique reflections	22,184 (2804)

Refinement statistics	
Resolution range (Å)	50.00 – 2.00
R_work_	0.177
R_free_	0.217
R.m.s.d (bonds)(Å)	0.009
R.m.s.d (angles)(°)	1.242
No of atoms	2405
No of waters	60

### Structure of FPPR and MPER

**Figure 3:**
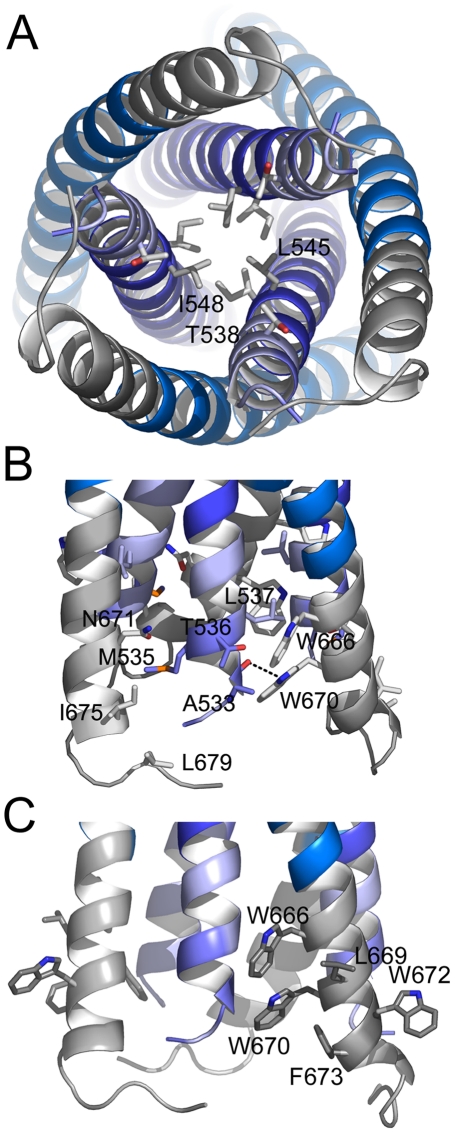
The FPPR-MPER regions are splayed apart. A) Close-up view from the bottom showing residue Leu 545 as the last coiled coil interacting residue of the HR1 core of gp41. The preceding potential heptad positions are Ala 541 and Thr 538. B) Close up view revealing mostly hydrophobic interactions between FPPR and MPER and only one hydrogen bond between the carbonyl of Ala and NE1 of Trp 670. C) Close-up of solvent exposed hydrophobic MPER residues.

Both FPPR and MPER extend HR1 and HR2 as continuous helices, but neither extension shows the regular knobs into holes packing reminiscent of classical coiled coils. Instead the FPPR region splays the inner core apart starting from Leu 545 (a position) (Fig. 3A). The distance between Arg 579 residues at the HR1 C-terminus is 12.5 Å while the one at the extreme N-terminus opens up to 22.7 Å (between Gly residues 531). As a consequence HR1 heptad positions are too far apart for interaction (Fig. 3A). The FPPR-MPER region is only stabilized by few hydrophobic contacts between adjacent chains, including interactions of Gly 531- Leu679, Ala 533- Trp 670, Met 535-Ile 675/Asn 671, Thr 536/Leu 537 - Trp 666 and one hydrogen bond between the carbonyl of Ala 533 and NE1 of Trp 670 (Fig. 3B). At position of MPER residue Asn 676, the N-terminus of FPPR-HR1 points towards the outside of the rod (Fig. 2A) facilitating fusion peptide (residues 512–530) membrane interaction or further refolding of FP with MPER and possibly TMR. Another striking feature of the structure is the solvent exposure of a stretch of hydrophobic MPER residues (Trp 666, Leu 669, Trp670, Trp 672, Phe 673) that generate a hydrophobic surface patch (Fig. S5).

Since the crystals were grown at a high MPD concentration, we tested the effect of MPD on the structure in solution. MPD does not change the overall helical content of gp41_528–683_, which is ∼90% in the absence and presence of high MPD concentrations (Fig. S3A). However, MPD reduced the T_m_ of gp41_528–683_ to 82.2°C (5% MPD) and 74.7°C (10% MPD) as well as that of the gp41_541–665_ core (Fig. S3B). Therefore, we cannot exclude the possibility that MPD might have destabilized the rod resulting in the ‘open’ structure (Fig. 3A) and FPPR and MPER might pack tighter in the absence of MPD.

### Comparison of MPER conformations

**Figure 4:**
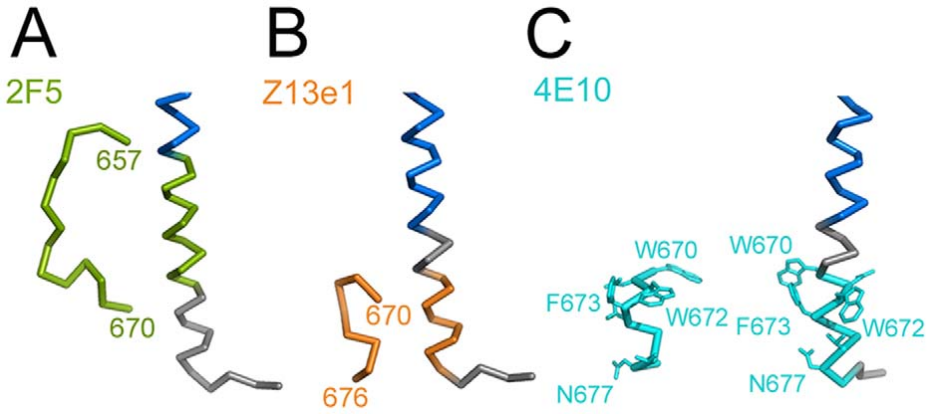
Comparison of MPER conformations. MPER conformations as determined in complex with broadly neutralizing antibodies (A) 2F5 [15], (B) Z13e1 [38] and (C) 4E10 [16] are shown in comparison to MPER within trimeric gp41. The corresponding MPER segments are colored equally and residues contacting the 4E10 Fab are shown as sticks. (blue, HR2).

The NMR structures of MPER peptides show kinked or straight helical conformations [17], [37], which superimpose partly with MPER present in the crystal structure (Fig. S6A and B). Three broadly neutralizing antibodies (nAb) target MPER and utilize diverse structural motifs for recognition. NAb 2F5 recognizes a beta-hairpin [15] and Z13e1 binds to a short kinked helix [38]. Both epitopes refold into a straight helix in the gp41 structure (Fig. 4A and B). The epitope of nAb 4E10 is helical [16]; although it is present and exposed in the gp41 crystal structure (Fig. 4C) nAb 4E10 does not interact with gp41_528–683_ (data not shown), due to clashes with the helical conformation of HR2. However, if we consider only MPER and its membrane orientation and dock the 4E10 structure onto its epitope, nAb 4E10 could present its heavy chain CDR3 loop implicated in bilayer interaction [14], [18] towards the membrane, lined up with the gp41 membrane embedded residues W678, W680 and Y681 (Fig. S7). The comparison of the peptide epitope structures and gp41 corroborate that nAbs 2F5 and Z13e1 block the refolding process of gp41 at early steps. In contrast the 4E10 epitope might be present throughout gp41 refolding from a native conformation as evident by its presence in the late fusion intermediate conformation.

## Discussion

Although the core structure of the HIV-1 fusion protein has been solved [4], [5], detailed structural information on the regions linking up to the membrane anchors (FPPR and MPER) has been lacking. We crystallized gp41_(528–683)_, which has a similar N- terminal end as a proteolytic fragment of HIV-2 gp41 [39] and N- and C-terminal ends as determined by peptide studies [40] and solved its structure. FPPR and most of MPER extend in a helical fashion from the gp41 core and interact with each other as indicated by peptide studies performed at pH 3.2 [40]. Although the interactions are mostly hydrophobic, they are not classical coiled-coil interactions. The TMR-juxtaposed region of MPER positions three aromatic side chains per monomer towards the membrane. We calculate that membrane insertion of these residues could induce membrane curvature in the outer leaflet of the viral lipid bilayer [35], which would facilitate fusion based on previous studies [36]. Membrane fusion models postulate that fusion proteins induce local bending of both bilayers into “nipples” projecting toward each other to reduce the energy requirement for initial stalk formation [22], [41], [42]. Bending on the target-cell side can be stabilized by insertion of the fusion peptide [43] or hydrophobic residues of fusion loops [26]. The present structure suggests that bending on the viral side may be stabilized by membrane-embedded MPER residues. We suggest that MPER membrane insertion may occur early during the conformational transition of gp41 and persist through the process of refolding. Alternatively this segment of MPER may adopt a straight helical conformation [37] in continuity with TMR in the final postfusion conformation. Such a continuous helical structure was observed for the linker sequences that connect the core SNARE complex to its TMRs [44].

The presence of FPPR and MPER splay the “membrane-anchor” end of the rod apart, which may be required to accommodate FP whose chain direction points to the exterior of the structure. The missing part of FP (residues 512–530) could thus contact the membrane and/or interact with the kinked membrane embedded MPER or with a straight helical MPER conformation. Since the thermostability measurements indicate that the MPD crystallization conditions could influence the stability of gp41 in solution, it is possible that FPPR and MPER pack tighter in the absence of MPD. We thus propose that the structure represents a late fusion intermediate state rather than the final postfusion conformation, although the latter possibility cannot be excluded.

MPER contains a number of hydrophobic residues, which are conserved in the majority of HIV and SIV isolates, namely Trp666, Trp672, Phe673 and Ile675. Single Ala mutations of these residues do not affect cell-cell fusion but reduce viral infectivity significantly [24]. Interestingly all residues are mostly exposed in the crystal structure and/or contribute to hydrophobic interactions with FPPR. Mutation of FPPR Leu537, which makes a hydrophobic contact with Trp666, in combination with mutations of conserved MPER residues Trp666 or Trp672 or Phe673 or Ile675, reduces virus infectivity further thus confirming the important interplay between FPPR and MPER during fusion [24]. Analysis showed that the defect of mutant Leu537-Trp666 is at the level of lipid mixing [24]. Another study demonstrated that mutations of the five conserved tryptophan residues (Trp666, Trp670, Trp 672, Trp678, Trp680) alone or in combination or deletion mutants within MPER affect syncytium formation thus supporting the importance of MPER for fusion [45]. Reduction in viral infectivity was also reported for pseudoviruses containing alanine mutations of hydrophobic MPER residues (Leu 669, Ile675, Leu679) exposed within MPER in addition to the conserved tryptophan residues [46]. The hydrophobic surface generated by the conserved MPER residues as shown here might induce clustering of several gp41 trimers at the site of fusion although the number of env trimers required for fusion is still debated [33], [34]. Such a function may be consistent with mutagenesis data showing that single tryptophan exchanges within MPER affect cell-cell fusion, while combinations of tryptophan mutations abrogate cell-cell fusion completely [45]. Thus mutagenesis of multiple tryptophans may reduce the hydrophobicity of the exposed patch sufficiently to affect the clustering function.

Six-helix bundle formation leads to fusion pore opening [20] and an intact six-helix bundle is required for its enlargement [47]. Since FPPR and MPER folding most likely follows six-helix bundle formation its hydrophobic patch may further support pore enlargement together with the essential role of TMRs [22], [25], [26]. This suggestion is in agreement with data on mutagenesis of all 5 tryptophan residues within MPER; these mutations do not affect fusion pore opening, but inhibit fusion pore expansion [23]. Finally the linker region that connects the SNARE complex with its TMR exposes a similar patch of hydrophobic residues [44] underlining functional similarities between viral fusion protein and SNARE-mediated membrane fusion processes.

Fusion proteins utilize the free energy released during their refolding to draw two membranes into close apposition and catalyze membrane fusion [26]. The thermostability measurement of the gp41 core compared to the crystal structure reveals a 12°C increase of the melting temperature, which can translate into an increase in ΔG that can be directly coupled to membrane fusion. Notably, folding of the complete SNARE complex versus the core produces a similar increase in T_m_ that can convert into energy for fusion [44].

MPER harbors the epitopes of three broadly neutralizing antibodies, 2F5, Z13e1 and 4E10. The epitopes of 2F5 and Z13e1 [15], [38] adopt a straight helical conformation, indicating that both antibodies neutralize by blocking the transition into the trimeric gp41 structure. In contrast the epitope of 4E10 [16] is still present and exposed, although nAb 4E10 does not interact with gp41_528–683_ due to clashes with the helical conformation of HR2. NAb 4E10 has a long CDR3 region that does not contact the epitope, but was proposed to interact with the membrane [16] based on its reactivity with lipids [14]. If we consider only the 4E10 epitope and the membrane embedded part of MPER, 4E10 could orient its CDR3 towards the membrane and insert its aromatic residues into the bilayer as required for neutralization [18]. Thus stabilization of a peptide in the conformation of the MPER as present in the crystal structure should prove useful to generate an immunogen capable of inducing 4E10-like antibody responses.

Based on the crystal structure we suggest the following extension to our picture of the fusion process. Receptor binding induced conformational changes exposes FP, which interacts and bends the target cell membrane. Concomitantly, TMR and MPER dissociate, potentially from a native MPER coiled-coil structure [48] and a few aromatic MPER residues insert into and bend the outer leaflet of the viral membrane. This then generates the functional epitope for nAb 4E10. Part of MPER stays membrane associated throughout the folding of the gp41 core that leads to fusion pore opening. Subsequently FPPR and the soluble part of MPER interact, releasing more energy for fusion. Alternatively, we cannot exclude the possibilities that (i) membrane insertion of MPER is already present in the native env trimer or (ii) that membrane insertion of MPER is not important for the generation of membrane curvature and exerts another role during the fusion process. Finally, although the conformational state of gp41 observed in the crystal structure is no longer targeted by neutralizing antibodies, the development of small molecules targeting the FPPR-MPER conformation could block further gp41 refolding required for membrane fusion.

## Materials and Methods

### Protein constructs

The gp41 proteins were assembled from different fragments of gp41 (Fig. S1): FPPR-HR1-HR2-MPER (Ser^528^ to Leu^581^ and Met^628^ to Lys^683^; gp41_528–683_), HR1-HR2 (Ala^541^ to Leu^581^ and Met^628^ to Lys^665^; gp41_541–665_). DNA sequencing and MALDI TOF Mass Spectrometry confirmed all constructs.

### Protein expression and purification

Fragments of HIV-1 gp41 HXB2 group M subtype B were amplified by standard PCR techniques and cloned either into pETM-MBP-1a (EMBL, Heidelberg), pETM-20 (thioredoxin fusion, EMBL, Heidelberg) or pET11 (His-tag). HR1 and HR2 containing constructs were N-terminally fused to the Flag-tag sequence (ASP-ASP-ASP-ASP-Lys) to improve solubility (Fig. S1).

Gp41_528–683_ and gp41_541–665_ fusion proteins were expressed in *E. coli* strain Rosetta 3 (DE3) (Strategene). Cells were grown to an OD_600 nm_ of 0.7 and induced with 1 mM IPTG at 37°C. After 2 hours cells were harvested by centrifugation, resuspended in buffer A (0.02 M Tris pH 8.0, 0.1 M NaCl) and pellets of HR1 and HR2 expressing bacteria were mixed before lysis. Notably, bacteria expressing HR2 were used in excess over HR1 expressing bacteria. The soluble fraction was loaded onto an amylose column (NEB) and eluted in buffer A with 0.01 M maltose. In order to remove fusion proteins, constructs were digested o. n. at 4 C° with TEV (Tobbacco Etch Protease) and the uncleaved material was removed by Ni^2+^ chromatography. Further purification was achieved by anionic exchange chromatography in buffer A. A final purification step included size exclusion chromatography on a superdex 200 column in buffer A.

### Crystallization, data collection and structure determination

Crystals of gp41_528–683_ were obtained by the vapor diffusion method in hanging drops mixing equal volumes of purified complex and reservoir solution (0.1 M citric acid pH 6, 60% MPD (v/v)). Crystals were improved by macroseeding; briefly crystals grown in the initial conditions (0.1 M citric acid pH 6, 60% MPD (v/v)) were transferred into a new drop equilibrated with 0.1 M citric acid pH 6, 56% MPD (v/v), 1.5% glycerol (v/v). Before data collection, crystals were flash frozen at 100 K using the same reservoir solution supplemented with 10% of glycerol (v/v).

A dataset was collected at the ESRF beam line ID14-EH4 at 100 K. The images were indexed with MOSFLM [49] and scaled with SCALA [50], [51]. The crystals were twinned and analysis with phenix.xtriage [52] revealed space group P6_3_ with twin fractions of 0.45 (Britton) and 0.47 (H test and Maximum likelihood test) and an associated twin law of h, -h-k, -l. The cell parameters are a = b = 57.42 Å, c = 182.76 Å, α = β = 90°, γ = 120°. The structure was solved by molecular replacement using the program Phaser [53] and the model of the gp41 core (PDB ID: 1AIK) by applying the twin law of h, -h-k, -l on the data, revealing 3 molecules in the asymmetric unit. The model was built manually with COOT [54] and refined with the program Phenix [52]. The final structure has an R_factor_ of 0.177 and R_free_ of 0.217 and good stereochemistry (Table 1). The most complete monomer contains gp41 residues 531–581 and gp41 residues 629–681 plus 5 N-terminal residues (624-DDDDK-628 derived from the Flag/enterokinase cleavage site sequence); this monomer was used to reconstruct the trimer by applying crystallographic symmetry. The second monomer contains residues 538–581 and 629–672 plus 5 N-terminal residues (residues 624-DDDDK-628); the third monomer contains residues 542–580 and 629–665 plus 5 N-terminal residues (residues 623-MDDDDK-628). All molecular graphics figures were generated with Pymol (http://www.pymol.org). Coordinates and structure factors have been deposited in the protein data bank with accession number 2×7r.

### CD spectroscopy

CD measurements were performed using a JASCO Spectropolarimeter equipped with a thermoelectric temperature controller. Spectra of each protein were recorded at 20°C in 1 nm steps from 190 to 260 nm in buffer A or buffer A supplemented with MPD as indicated. Spectra were recorded at 222 nm using a bandwidth of 4 nm and averaging time of 4 sec per step. For thermal denaturation experiments, the ellipticity was recorded at 222 nm with 1°C steps from 20° to 100°C with an increment of 80°C h^−1^ and an averaging time of 30 s/step. Since the unfolding of gp41_528–683_ was not reversible, two more spectra were recorded with increments of 40°C h^−1^ and 120°C h^−1^, which resulted in comparable Tms, indicating that the system was in equilibrium. For data analysis, spectra were corrected for the baseline (recorded with buffer) and the raw ellipticity values were converted to mean residue ellipticity. Thermal melting (T_m_) points were calculated with a Boltzmann sigmoid fit using the program OriginLab.

### Physical model

The effective shape of a membrane embedding domain consisting of the gp41 hydrophobic residues was approximated by a short cylindrical rod of 16 Å in length and 7 Å in diameter, shallowly inserted up to a 5 Å depth into the outer membrane monolayer (the insertion volume constituting 468.9 Å^3^). According to the previously developed model of membrane bending by hydrophobic insertions [35] the effective spontaneous curvature of such an insertion equals 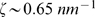. The overall membrane curvature generated by the insertions is proportional to their area fraction in the membrane plane whose maximal value is limited by a dense packing of the proteins on the membrane surface. For a gp41 trimer the area of each of the three inserted side chains is 16 Å×7 Å = 112 Å^2^, while the total area of the trimer projection on the membrane plane is determined by the dimensions of the ectodomains and constitutes, approximately, 800 Å^2^. Taking into account these numbers, we obtain that a maximal area fraction of the gp41 hydrophobic insertions is 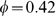 which results in a total radius of curvature 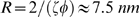.

## Supporting Information

Figure S1 Schematic drawing of gp41 and of the expression constructs employed to assemble gp41. TRX, thioredoxin fusion protein; MBP, maltose binding protein; EK, enterokinase cleavage site and flag sequence.
(4.09 MB TIF)

Figure S2 Size exclusion chromatography (SEC) analysis of gp41. Gp41_528-683_ elutes from a S200 column at ∼12 ml similar to the elution profile of the marker protein aldolase (158 kDa) consistent with its elongated shape. Notably the previously determined trimeric core of gp41, gp41_(541–665)_ elutes later at 14.5 ml consistent with a shorter trimeric rod [28] [5]. The inset shows the SDS-PAGE analysis of the complex formed by gp41 peptides containing residues flag-528 to 581 and residues flag-628 to 683.
(4.60 MB TIF)

Figure S3 Circular dichroism analysis of gp41 constructs. Spectra were recorded at room temperature and normalized to mean residue ellipticity. The presence of MPD in the buffer is indicated in % (MPD). (A) The helical content of gp41_528–683_ was calculated to be 89%. This corresponds well with the crystal structure, revealing 20 residues out of 126 residues disordered or in a non-helical conformation. Increasing concentrations of MPD (5, 10 and 40%) did not change the overall helical content. (B) Since the Tm of gp41_528–683_ was 87.6°C, we tested whether high MPD concentrations required for crystal formation might have affected the interactions within gp41_528–683_. This showed that MPD reduced the Tm of gp41_528–683_ to 82.2°C (5% MPD) and 74.7°C (10% MPD) as well as that of the gp41_541–665_ core.
(5.41 MB TIF)

Figure S4 Model of gp41_528–683_ membrane association. Residues Trp 678, Trp 680 and Tyr 681 insert their side chains into one leaflet of the bilayer, thus inducing local membrane curvature. The position of the TMR is represented by one TMR (green).
(3.06 MB TIF)

Figure S5 Surface representation of trimeric gp41_528–683_. Exposed hydrophobic residues are colored in green. Note that the MPER region forms an extended hydrophobic surface patch.
(4.61 MB TIF)

Figure S6 Comparison of the trimeric gp41 MPER with conformations of MPER peptides. Overlay of the Cα atoms of the NMR MPER peptide structures (A) (pdb entry 2PV6; (ELDKWASLWNWFNITNWLWYIK) [17] (shown in cyan) and (B) pdb entry 1JAV (KWASLWNWFNITNWLWYIK) [37] (shown in green). Residues recognized by nAb 4E10 are indicated.
(2.38 MB TIF)

Figure S7 Overlay of Cα atoms of MPER present in the crystal structure with the 4E10 peptide complex structure [16]. Side chains of membrane-embedded MPER are shown as well as hydrophobic side chains of the 4E10 heavy chain CDR3 region (shown in salmon). W100 and L100C are oriented in a way that permits membrane insertion as postulated [18]. W100B whose orientation is determined by a water mediated polar contact could contribute to membrane interaction upon flipping sideward.
(2.30 MB TIF)
