# Supplementary figures and images for: Crystal Structure of HIV-1 gp41 Including Both Fusion Peptide and Membrane Proximal External Regions
Source: PLoS Pathog. 2010 May 6;6(5):e1000880. doi: 10.1371/journal.ppat.1000880 (PMC2865522; doi:10.1371/journal.ppat.1000880)

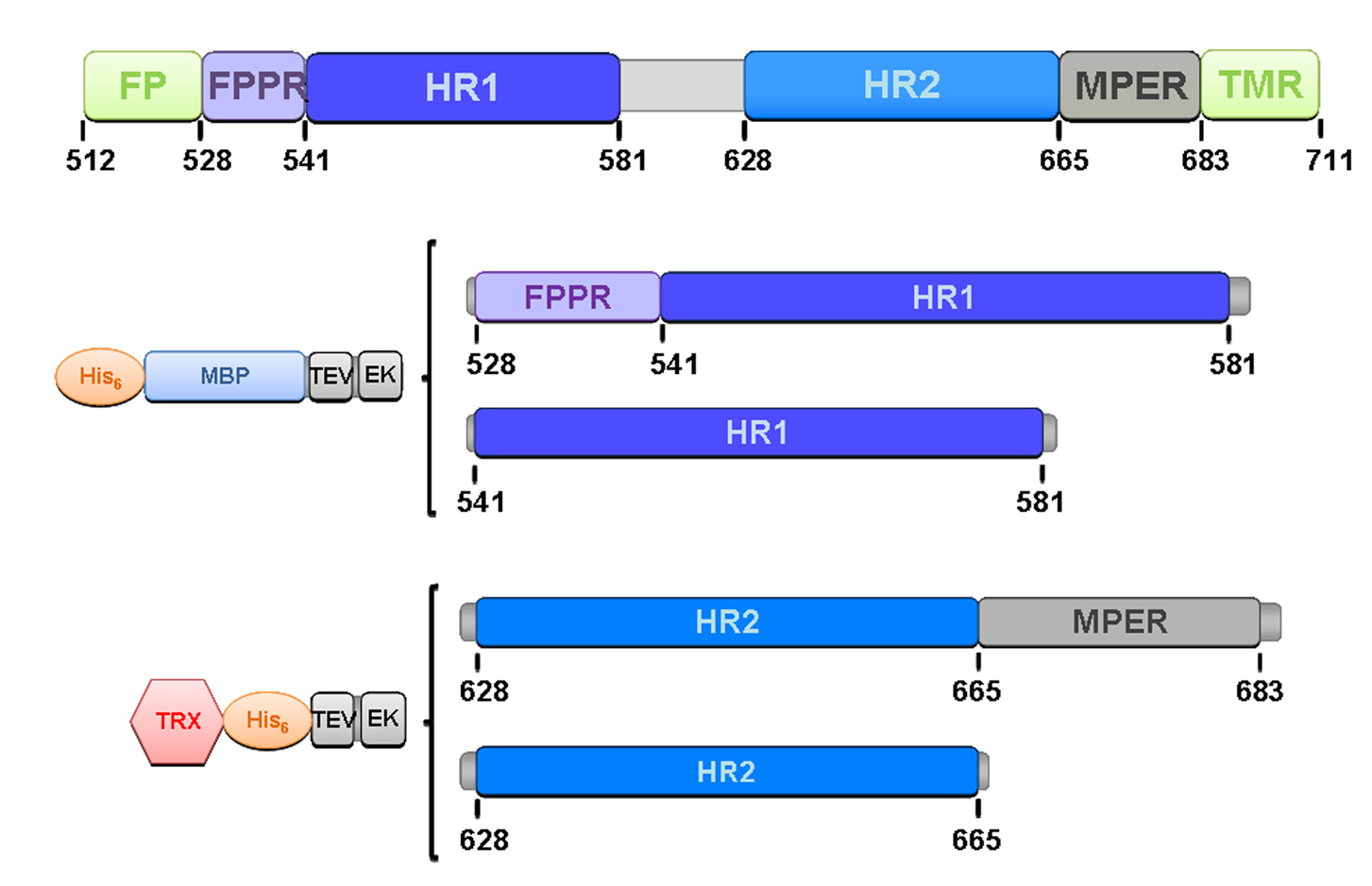

Supplement: Figure S1 — Schematic drawing of gp41 and of the expression constructs employed to assemble gp41. TRX, thioredoxin fusion protein; MBP, maltose binding protein; EK, enterokinase cleavage site and flag sequence. (4.09 MB TIF) [file ppat.1000880.s001.tif]

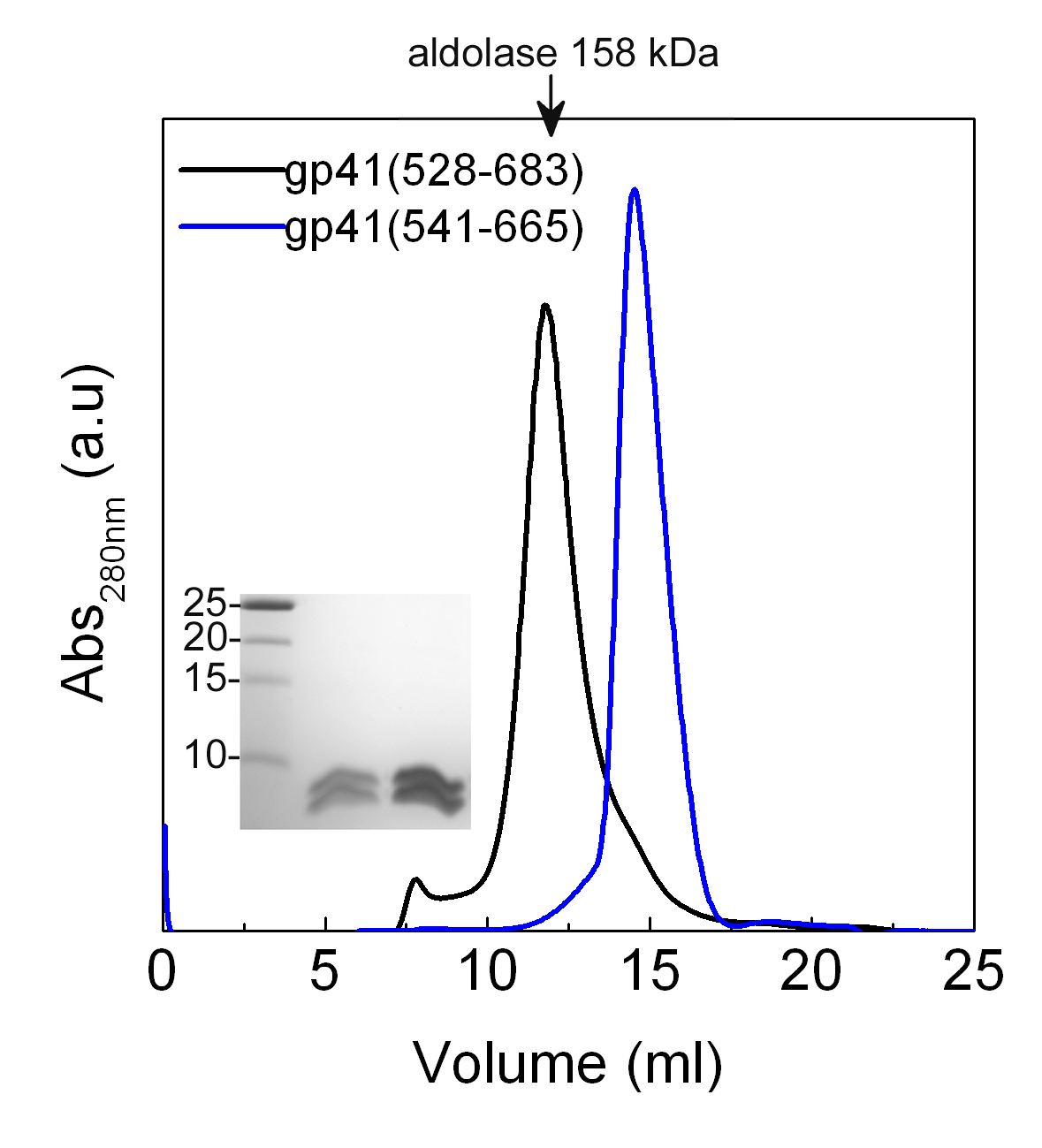

Supplement: Figure S2 — Size exclusion chromatography (SEC) analysis of gp41. Gp41528-683 elutes from a S200 column at ∼12 ml similar to the elution profile of the marker protein aldolase (158 kDa) consistent with its elongated shape. Notably the previously determined trimeric core of gp41, gp41(541–665) elutes later at 14.5 ml consistent with a shorter trimeric rod [28] [5]. The inset shows the SDS-PAGE analysis of the complex formed by gp41 peptides containing residues flag-528 to 581 and residues flag-628 to 683. (4.60 MB TIF) [file ppat.1000880.s002.tif]

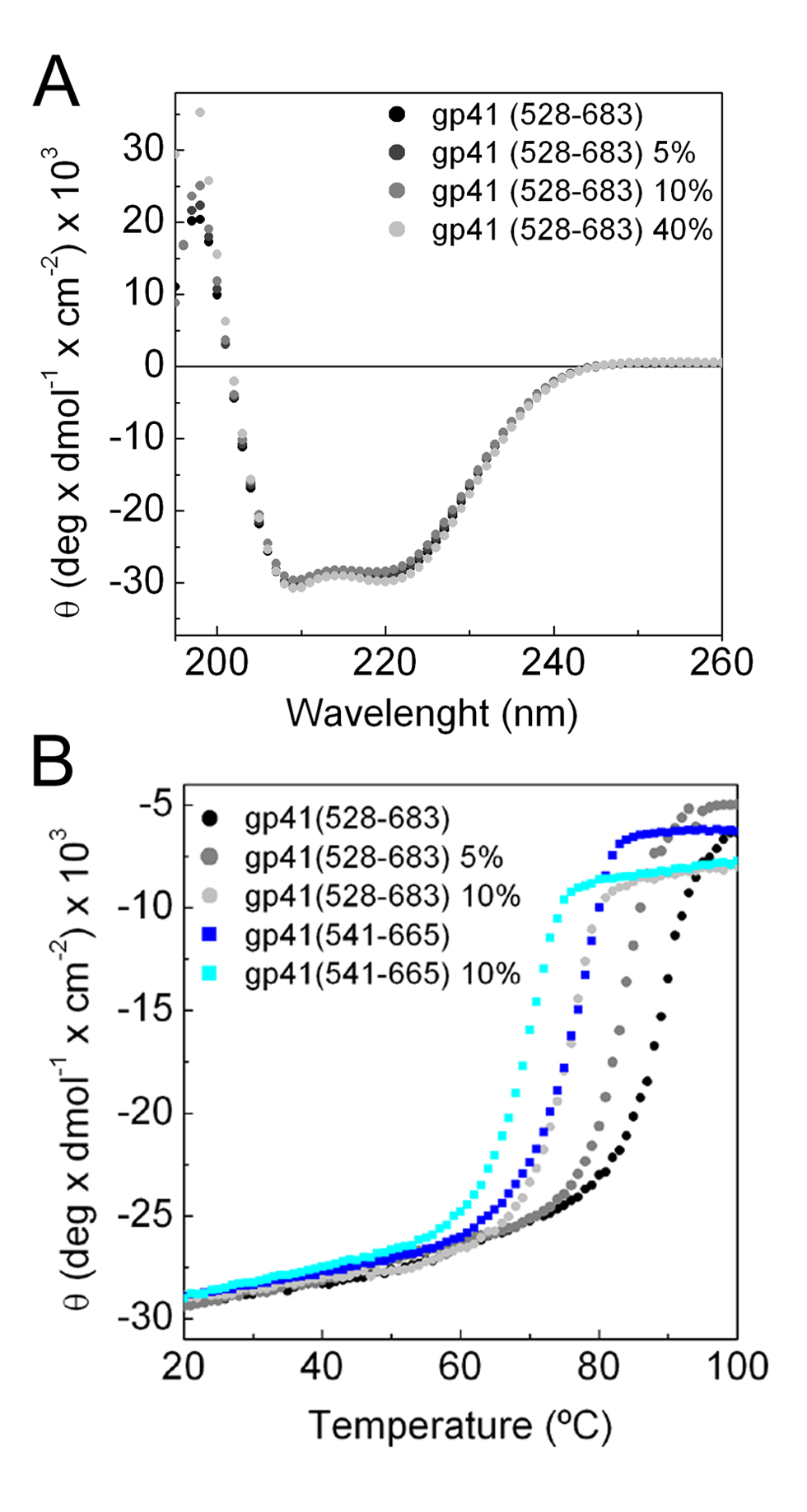

Supplement: Figure S3 — Circular dichroism analysis of gp41 constructs. Spectra were recorded at room temperature and normalized to mean residue ellipticity. The presence of MPD in the buffer is indicated in % (MPD). (A) The helical content of gp41528–683 was calculated to be 89%. This corresponds well with the crystal structure, revealing 20 residues out of 126 residues disordered or in a non-helical conformation. Increasing concentrations of MPD (5, 10 and 40%) did not change the overall helical content. (B) Since the Tm of gp41528–683 was 87.6°C, we tested whether high MPD concentrations required for crystal formation might have affected the interactions within gp41528–683. This showed that MPD reduced the Tm of gp41528–683 to 82.2°C (5% MPD) and 74.7°C (10% MPD) as well as that of the gp41541–665 core. (5.41 MB TIF) [file ppat.1000880.s003.tif]

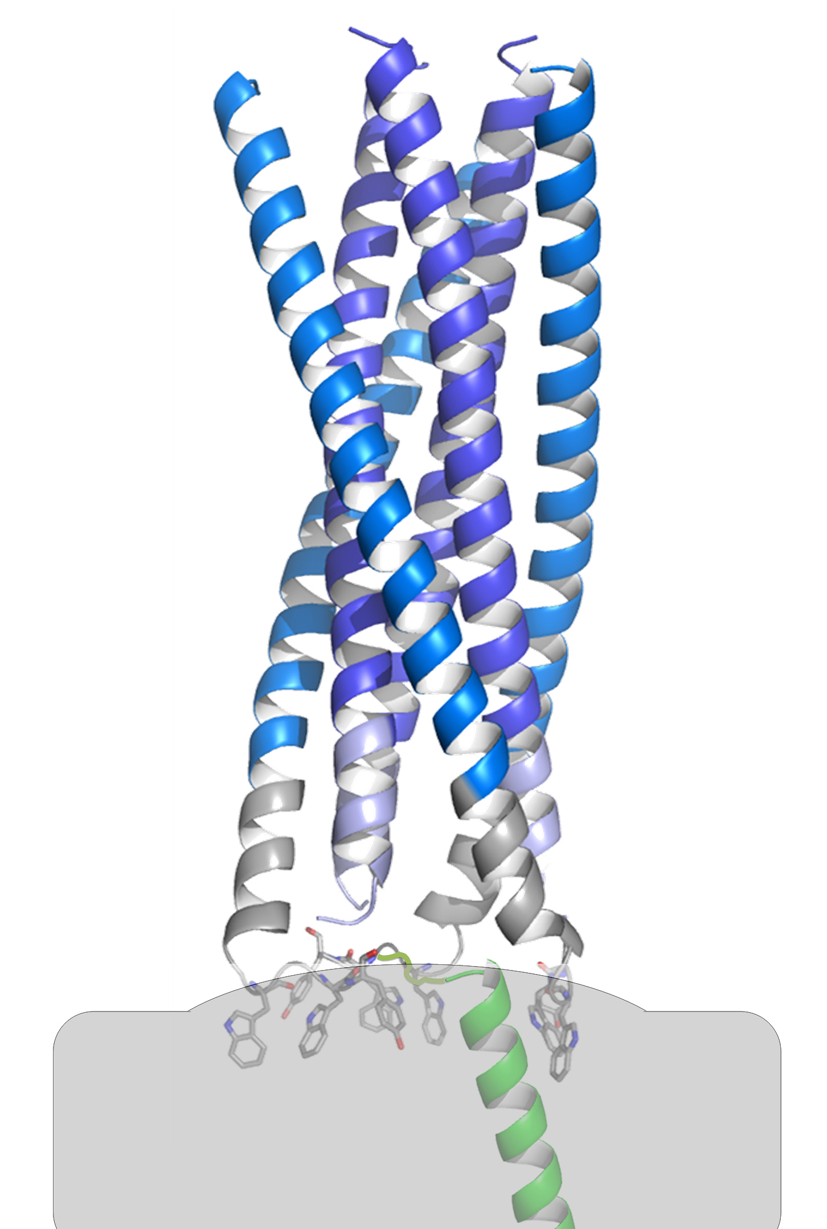

Supplement: Figure S4 — Model of gp41528–683 membrane association. Residues Trp 678, Trp 680 and Tyr 681 insert their side chains into one leaflet of the bilayer, thus inducing local membrane curvature. The position of the TMR is represented by one TMR (green). (3.06 MB TIF) [file ppat.1000880.s004.tif]

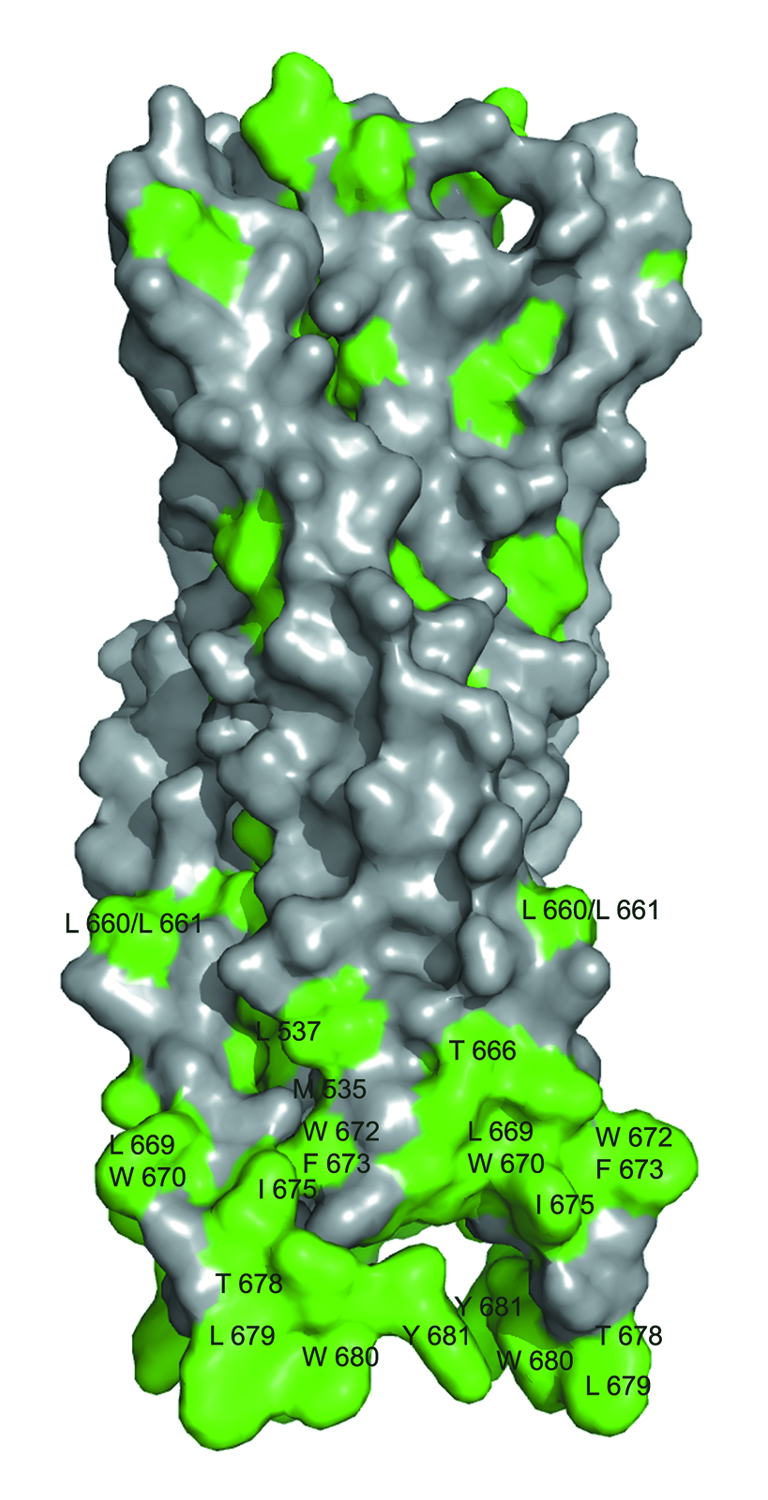

Supplement: Figure S5 — Surface representation of trimeric gp41528–683. Exposed hydrophobic residues are colored in green. Note that the MPER region forms an extended hydrophobic surface patch. (4.61 MB TIF) [file ppat.1000880.s005.tif]

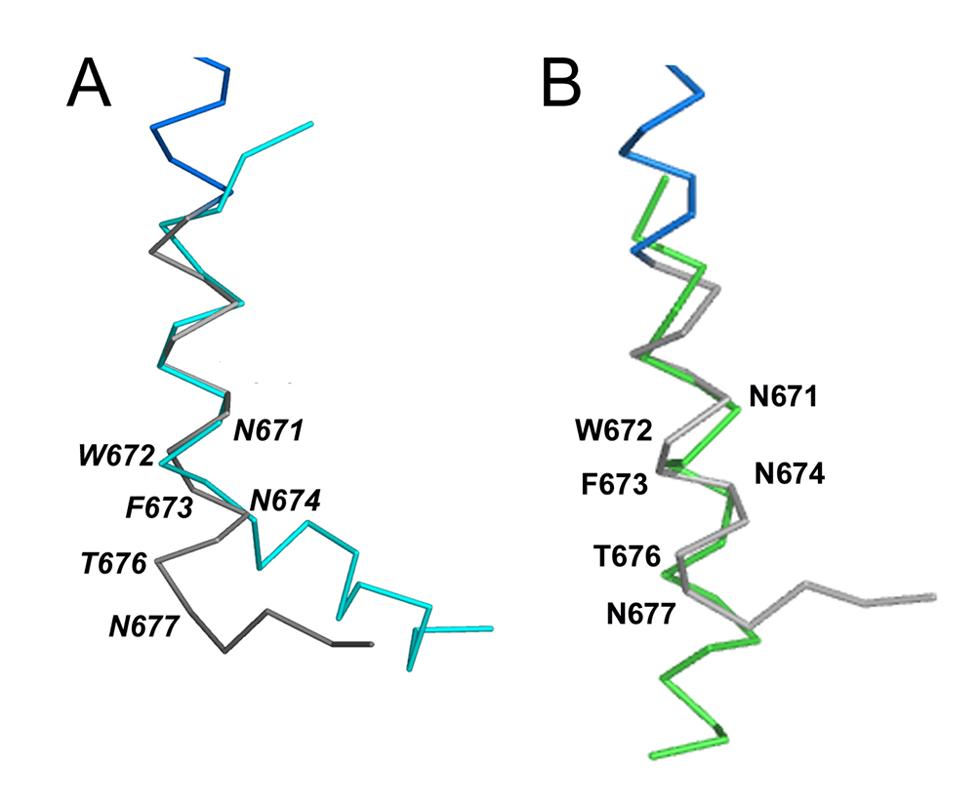

Supplement: Figure S6 — Comparison of the trimeric gp41 MPER with conformations of MPER peptides. Overlay of the Cα atoms of the NMR MPER peptide structures (A) (pdb entry 2PV6; (ELDKWASLWNWFNITNWLWYIK) [17] (shown in cyan) and (B) pdb entry 1JAV (KWASLWNWFNITNWLWYIK) [37] (shown in green). Residues recognized by nAb 4E10 are indicated. (2.38 MB TIF) [file ppat.1000880.s006.tif]

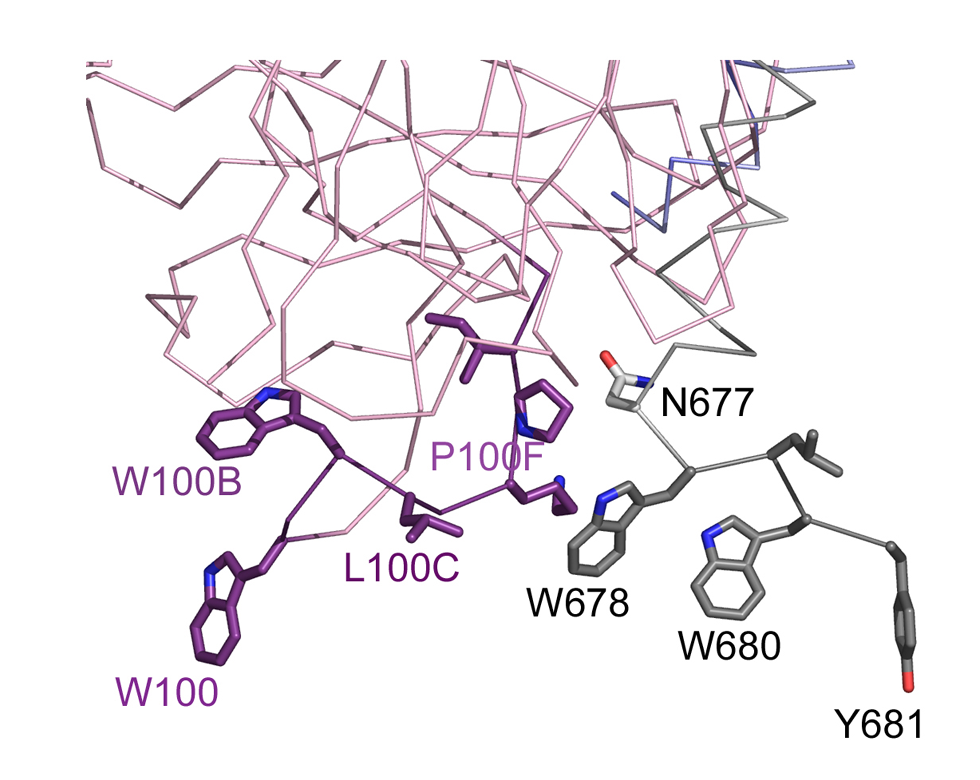

Supplement: Figure S7 — Overlay of Cα atoms of MPER present in the crystal structure with the 4E10 peptide complex structure [16]. Side chains of membrane-embedded MPER are shown as well as hydrophobic side chains of the 4E10 heavy chain CDR3 region (shown in salmon). W100 and L100C are oriented in a way that permits membrane insertion as postulated [18]. W100B whose orientation is determined by a water mediated polar contact could contribute to membrane interaction upon flipping sideward. (2.30 MB TIF) [file ppat.1000880.s007.tif]
